# Fungal Disease Prevention in Seedlings of Rice (*Oryza sativa*) and Other Grasses by Growth-Promoting Seed-Associated Endophytic Bacteria from Invasive *Phragmites australis*

**DOI:** 10.3390/microorganisms6010021

**Published:** 2018-03-08

**Authors:** Satish K. Verma, Kathryn L. Kingsley, Marshall S. Bergen, Kurt P. Kowalski, James F. White

**Affiliations:** 1 Department of Plant Biology, Rutgers University, New Brunswick, NJ 08901, USA; kathryn.l.kingsley@gmail.com (K.L.K.); bergenm@scarletmail.rutgers.edu (M.S.B.); 2 Centre of Advanced Study in Botany, Banaras Hindu University, Varanasi, UP 221004, India; 3 U.S. Geological Survey, Great Lakes Science Center, 1451 Green Road, Ann Arbor, MI 48105-2807, USA; kkowalski@usgs.gov

**Keywords:** antifungal activity, biocontrol, disease suppression, seedling development

## Abstract

Non-cultivated plants carry microbial endophytes that may be used to enhance development and disease resistance of crop species where growth-promoting and protective microbes may have been lost. During seedling establishment, seedlings may be infected by several fungal pathogens that are seed or soil borne. Several species of *Fusarium*, *Pythium* and other water moulds cause seed rots during germination. *Fusarium* blights of seedlings are also very common and significantly affect seedling development. In the present study we screened nine endophytic bacteria isolated from the seeds of invasive *Phragmites australis* by inoculating onto rice, Bermuda grass (*Cynodon dactylon*), or annual bluegrass (*Poa annua*) seeds to evaluate plant growth promotion and protection from disease caused by *Fusarium oxysporum*. We found that three bacteria belonging to genus *Pseudomonas* spp. (SLB4-*P. fluorescens*, SLB6-*Pseudomonas* sp. and SY1-*Pseudomonas* sp.) promoted seedling development, including enhancement of root and shoot growth, and stimulation of root hair formation. These bacteria were also found to increase phosphate solubilization in in vitro experiments. *Pseudomonas* sp. (SY1) significantly protected grass seedlings from *Fusarium* infection. In co-culture experiments, strain SY1 strongly inhibited fungal pathogens with 85.71% growth inhibition of *F. oxysporum*, 86.33% growth inhibition of *Curvularia* sp. and 82.14% growth inhibition of *Alternaria* sp. Seedlings previously treated with bacteria were found much less infected by *F. oxysporum* in comparison to non-treated controls. On microscopic observation we found that bacteria appeared to degrade fungal mycelia actively. Metabolite products of strain SY1 in agar were also found to inhibit fungal growth on nutrient media. *Pseudomonas* sp. (SY1) was found to produce antifungal volatiles. Polymerase chain reaction (PCR) amplification using specific primers for pyrrolnitirin synthesis and HCN (hydrogen cyanide) production suggested presence of genes for both compounds in the genome of SY1. HCN was detected in cultures of SY1. We conclude that microbes from non-cultivated plants may provide disease protection and promote growth of crop plants.

## 1. Introduction

Protection of crop plants from pathogens and improvement of plant productivity are critical in the context of increasing demand for food to support the growing world population. Rice is an important staple food crop worldwide. During seedling establishment, rice may be infected by fungal pathogens [1]. Several species of *Fusarium*, *Pythium* and other water molds cause rice seed rot during germination. Fungal pathogens including *Fusarium* spp., *Curvularia* spp. and *Rhizoctonia solani* also cause blights in rice seedlings. These pathogens are mostly seed-borne and affect seed germination and development [1]. Rice sheath rot is also one of the most destructive diseases caused by several pathogens including *Fusarium* spp. and *Sarocladium oryzae* and are transmitted by seeds from generation to generation [2]. Currently, many cultural and non-cultural practices are being used to protect plants from fungal pathogens; however it is difficult to completely eliminate pathogens from fields [3]. A common practice to fight pathogens is use of chemical pesticides, which are not species specific, and cause deterioration of the beneficial microbial community [3]. Furthermore, pesticide use causes problems in the entire ecosystem. Presently, a sustainable approach to protect crops from pathogens is to use biocontrol agents (BCA). Plant microbiomes, including endophytes and epiphytes, are good examples of indigenous biocontrol agents [4,5]. Non-pathogenic symbiotic microbes including bacteria and fungi allow plants to adapt to the environment, and they also provide defense from biotic and abiotic stresses [6,7,8,9]. Endophytic or rhizospheric BCA protect plants from pathogens directly by producing antimicrobial metabolites, enzymes and antifungal lipopeptides [5,10,11], or indirectly by inducing plant immunity through induced systemic resistance [12], or by competitive nutrient mobilization to the host plant [5]. Endophytic microbes have also been found to induce up-regulation of defense genes in the host plant [11,13]. Some species of *Bacillus* and *Pseudomonas* have been reported to produce hydrogen cyanide (HCN) that inhibits fungal growth. Antifungal lipopeptides have also been reported from *Pseudomonas* spp., *Bacillus* spp. and some other bacteria [11,14]. 

## 2. Materials and Methods

### 2.1. Plant Materials

Rex rice (*Oryza sativa* L.) and Bermuda grass (*Cynodon dactylon* L.) seeds were procured from Hancock Farm & Seed Company (Dade City, FL, USA) and stored at 4 °C in a refrigerator. Seeds of annual bluegrass (*Poa annua* L.) were obtained from Dr. David Huff in the Department of Plant Science at Pennsylvania State University. 

### 2.2. Bacterial Isolates

A total of nine seed-associated bacterial isolates including strains *Pseudomonas* sp. strain West9 (GenBank KX650874), *P. fluorescens* strain SLB4 (GenBank KX665565), *Pseudomonas* sp. strain SY1 (GenBank MG197704), *Pantoea* sp. strain SY4 (GenBank MG746600), *Pseudomonas* sp. strain SY5 (GenBank MG197705), *Pseudomonas* sp. strain SLB6 (KX650502), *Pseudomonas* sp. strain RiY3 (KX650500) and *Pseudomonas* sp. strain ROLB13w (KX650501) from *Phragmites australis* [9] were screened for growth promotion and fungal infection susceptibility on rice, annual bluegrass and Bermuda grass seedlings.

### 2.3. Molecular Identification of Bacteria

For molecular identification of isolates that were not previously identified [9], total genomic DNA was extracted using a DNA extraction kit (Qiagen, Germantown, MD, USA) and the 16S ribosomal DNA (rDNA) sequences were amplified using primers (16SF, 16SR). The PCR products were purified and sent to Genewiz Inc. (South Plainfield, NJ, USA) for sequencing. The sequences were searched by basic local alignment (BLAST) on the National Center for Biotechnology Information GenBank database to find the closest matches. Sequences were submitted to GenBank. 

### 2.4. Cleaning of Rice, Bermuda Grass and Annual Bluegrass Seeds 

Rice seeds were disinfected by treatment with 4% NaOCl for 1 h with constant agitation and washed with sterile water. Seeds were then dipped in 95% ethanol for 3–5 min. Bermuda grass and annual bluegrass seeds were treated with 4% NaOCl for 20–25 min. To remove traces of NaOCl, all seeds were washed several times with sterile double-distilled water. 

### 2.5. Seedling Growth Promotion Experiments in Agarose Plates and Magenta Boxes

Disinfected seeds of rice and Bermuda grass were inoculated with overnight cultures of all nine bacterial isolates (West9, Microbac, SLB4, SY1, SY4, SY5, SLB6, RiY3 and ROLB13y) with 10^6^–10^8^ cells mL^−1^ suspension for two hours in Petri plates (5 mL per 50 seeds for rice and 1 mL per 50 seeds for Bermuda grass) and plated on 0.7% agarose plates (6 seeds of rice and 20 seeds of Bermuda grass on each plate). After seven days of incubation, growth parameters including geotropic response, root and shoot lengths and root hair formation were recorded. Geotropic response was determined as a percentage by counting the number of seedlings showing downward growth of seedling roots/total seedlings × 100. The most active three isolates including SLB4, SLB6 and SY1 on rice seedlings were also used in magenta box experiments, where boxes each contained 15 grams of potting mix (peat, sand and perlite in 2:1:1 ratio) and 40 mL of sterile water. Tests with each microbe were set up in triplicate with 20 seeds in each box. A control included magenta boxes containing potting mix with surface-disinfected seeds that had not been inoculated with bacteria. 

### 2.6. Phosphate Solubilization Activity 

A phosphate solubilization assay was performed by using Pikovskaya agar [15]. For this assay, one-day-old cultures of all the bacteria were streaked onto Pikovskaya agar medium and after 5 days of incubation, transparent zones around bacterial colonies were noted as positive for phosphate solubilization. 

### 2.7. Antifungal Activity of Strain SY1 

Bacteria were tested for antagonism against three soil borne fungal pathogens including *Fusarium oxysporum*, *Curvularia* sp. and *Alternaria* sp. The experiment was done with the dual culture technique on PDA plates and the percentage inhibition of the growth of the pathogens was calculated using the following formula: % inhibition = (R_1_ − R_2_/R_1_) × 100 [16], where R_1_ is the radial distance grown by the pathogen on the control plate and R_2_ is the radial distance grown by pathogens against tested bacterium streaked onto the same plate.

### 2.8. Antifungal Activity Assays: Agar Diffusion, Volatiles and HCN Production 

The strain SY1 was grown on potato dextrose agar (PDA) in Petri dishes for 5–7 days. Using a cork borer (1-cm diameter) agar was cut from the plate, avoiding bacterial colonies, and placed opposite to fungal pathogens *F. oxysporum*, *Curvularia* sp. and *Alternaria* sp. on PDA plates. Inhibition of radial growth was assessed after 5–7 days of incubation. 

For volatile antifungal compound assessment, bacteria and fungi were inoculated onto PDA Petri plates opposite one another, and a wedge of medium 2 cm wide was removed between the organisms. Plates were sealed with Parafilm^®^ and incubated under laboratory conditions for 7 days. After that time, inhibition in radial growth of the fungi was assessed.

A qualitative assay for HCN production by bacterial strain SY1 was carried out using the method described by Bakker and Schipper [17]. The bacterium was streaked onto King’s B medium, and sterile filter paper saturated with picric acid solution (2.5 g of picric acid; 12.5 g of Na_2_CO_3_, 1000 mL of distilled water) was fixed in the upper top cover of three Petri plates. Petri plates were sealed with Parafilm^®^ and incubated under laboratory ambient conditions for three days. A change in color of the filter paper from yellow to brown was recorded as a positive indication of HCN production.

### 2.9. Strain SY1 Antifungal Activity on Rice, Bermuda Grass and Annual Bluegrass Seedlings 

Disinfected rice, Bermuda grass and annual bluegrass seeds were treated with endophytic bacteria, incubated overnight and then inoculated with a water suspension of the fungal pathogen *F. oxysporum* (10^4^–10^6^ cells mL^−1^) produced on PDA plates, along with bacteria free controls for 2 h. Six rice seeds were placed onto each agarose plate, and 20 seeds were placed into magenta boxes containing previously sterilized potting mix. Treatments were set up in triplicate. Bermuda grass and annual bluegrass seeds were also placed onto agarose plates with similar treatments. Daily seedling roots were observed microscopically for *F. oxysporum* infection in root tissues along with controls over seven days of incubation. 

Infection experiments were done using seedlings of rice, Bermuda grass and annual bluegrass on 0.7% agarose plates. Seeds were surface disinfected as described previously, then treated with sterile water (control) or a suspension of bacterium SY1; with both treated with a suspension of conidia and mycelium of *F. oxysporum* (10^4^–10^6^ cells mL^−1^) produced on PDA plates. Seeds were plated onto the surface of 0.7% agarose and incubated for five days under laboratory ambient conditions, after which the percentages of seedlings bearing infection by the fungus were determined. 

### 2.10. Reactive Oxygen and SYTO13^®^ Staining to Visualize Bacteria

Agarose-penetrating roots of seven-day-old seedlings of rice and Bermuda grass grown on 0.7% agarose were stained by flooding plates with 2.5 mM diaminobenzidine tetrachloride (DAB; Sigma-Aldrich, Saint Louis, MO, USA) for 15 h. Roots were then washed with sterile water and counter-stained with aniline blue stain prior to observation under a light microscope. DAB enables visualization of reactive oxygen (H_2_O_2_) produced around inter- and intracellular bacteria [18]. SYTO13^®^ fluorescent stain was also used to visualize SY1 in tissues of rice and Bermuda grass seedling roots. 

### 2.11. Screening for Antifungal Genes in Strain SY1 

Genes involved in major antibiotic production were detected by PCR using gene-specific primers. Eight pairs of primers were used to amplify target genes including: 1. phenezine (primer PHZ1: GGCGACATGGTCAACGG3; PHZ2 CGGCTGGCGGCGTATTC), 2. phzCD–phenezine-1-carboxylic acid (primer PCA2a: TTGCCAAGCCTCGCTCCAAC; primer PCA3b: CCGCGTTGTTCCTCGTTCAT), 3. phzE–phenezine (primer phzEf: GAAGGCGCCAACTTCGTYATCAA; primer phzEr: GCCYTCGATGAAGTACTCGGTGTG), 4. phzF–phenezine (primer Ps up1 ATCTTCACCCCGGTCAACG; primer Ps low1: CCRTAGGCCGGTGAGAAC) 5. phlD–2,4-Diacetylphloroglucinol (primer Phl2a: GAGGACGTCGAAGACCACCA; primer Phl2b: ACCGCAGCATCGTGTATGAG), 6. prnD–pyrrolnitrin (primer PRND1: GGGGCGGGCCGTGGTGATGGA; primer PRND2: YCCCGCSGCCTGYCTGGTCTG), 7. hcnBC–hydrogen cyanide (primer Aca: ACTGCCAGGGGCGGATGTGC; primer Acb: ACGATGTGCTCGGCGTAC) and 8. PLTC-pyoleutirin gene (primer PLTC1: AACAGATCGCCCCGGTACAGAACG; primer PLTC2: AGGCCCGGACACTCAAGAAACTCG). These primers have been used in previous studies to amplify genes from *Pseudomonas* spp. [19,20,21]. Oligonucleotide primers were synthesized by Invitrogen, USA. The PCR reaction contained 22.5 µL super mix (Invitrogen), 0.5 µL each of forward and reverse primer (10 µM) and 1.5 µL of template (10 ng µL^−1^). The PCR was set up with initial denaturation at 94 °C for 5 min followed by 30 cycles (94 °C for 1 min, 58 °C for 30 s and 72 °C for 1 min) with final extension at 72 °C for 10 min. Each amplification product was run on a 1% agarose gel and visualized under UV light. 

### 2.12. Extraction and MALDI-TOF Analysis for Lipopeptides

Bacteria were grown in 1 L lysogeny broth with shaking at 200 rpm for 4 days, and cell supernatant was collected by centrifugation at 5000 rpm for 15 min at 4 °C. Concentrated HCl was added to the supernatant to reduce the pH to 2 and incubated overnight at 4 °C. A precipitate was collected after centrifugation at 8000 rpm at 4 °C for 15 min. The pellet was dissolved in methanol and filtered with a 0.45 µm PTFE polymer membrane filter to remove cell debris or larger particles and then concentrated by a vacuum evaporator at 30 °C. The final methanolic extract was dried by lyophilization and dissolved in methanol. Molecular mass determination of potential lipopeptides was done by Matrix Assisted Laser Desorption/Ionization (MALDI-TOF) analysis. To accomplish this, a sample (100 µg/µL) was diluted 10× with alpha-cyano-4-hydroxycinnamic acid (CHCA) in 50% acetonitrile and 0.1% trifluoroacetic acid (TFA). Data were acquired at reflector positive mode from 800 to 4000 *m*/*z*. The MALDI-TOF analysis was performed at Robert Wood Johnson Medical School, Rutgers University, Piscataway, NJ, USA.

## 3. Results

### 3.1. Effect of Bacteria on Rice and Bermuda Grass Seedling Growth and Development

**Figure 1:**
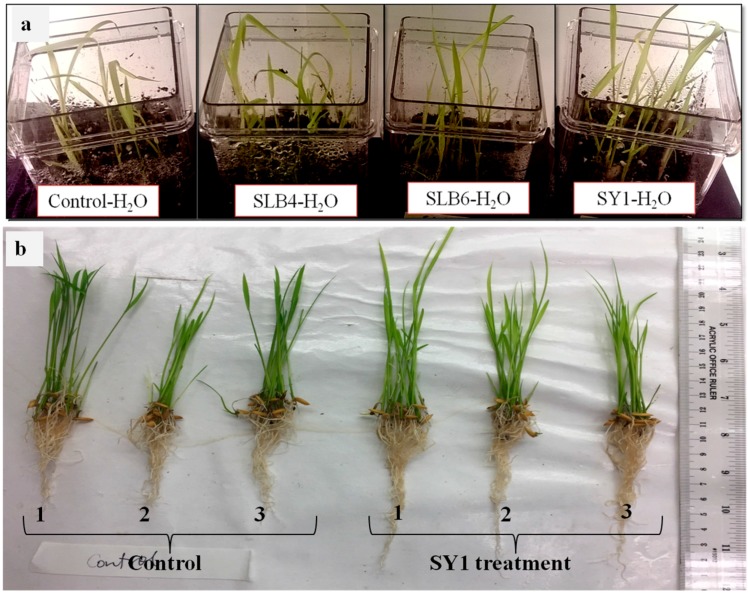
(**a**) Rice seedlings inoculated with bacteria (H_2_O only or strains SLB4, SLB6 and SY1) in magenta boxes containing potting mix. (**b**) Seedlings showing differences in root and shoot lengths between control and SY1-treated rice seedlings after 15 days grown in potting mix.

Nine seed-associated bacteria from *Phragmites australis* were screened for promotion of rice seedling growth, and five were screened on Bermuda grass seedlings as described in Table 1 and Table 2. Out of the nine, five isolates including West9, SLB4, Microbac, SY1 and SLB6 promoted geotropic response in rice seedlings roots. These isolates were also found to promote root and shoot length growth, root hair formation and root branching. Isolates including SLB4, SLB6 and SY1 were found to be most active (Table 1, Figure 1). In Bermuda grass, the four isolates West9, SLB4, SY1 and SLB6 induced seedling growth and root hair formation (Table 2). 

### 3.2. Phosphate Solubilization 

All isolates were found to be positive for phosphate solubilization activity (Table 1). 

### 3.3. Antifungal Activity of Bacteria 

**Figure 2:**
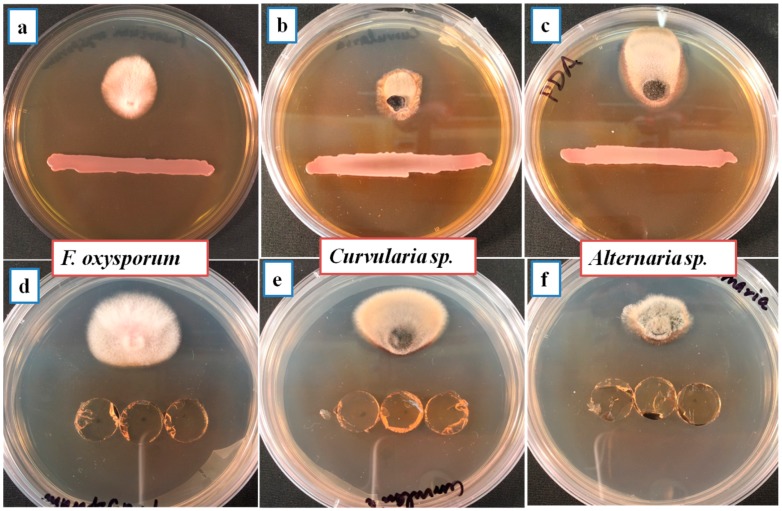

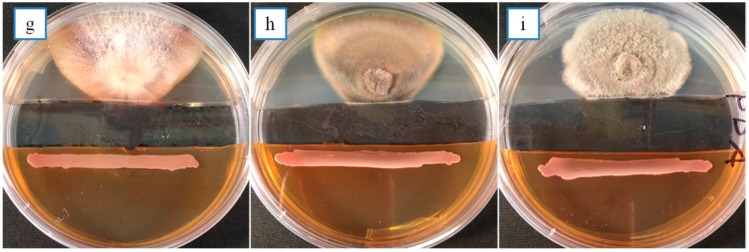
Antifungal activity of SY1 (*Pseudomonas* sp.) against *F. oxysporum*, *Curvularia* sp. and *Alternaria* sp.: Where first set (**a**–**c**) are antagonism in dual culture method; second set (**d**–**f**) are antifungal by agar diffusion (using bacterial-free plugs of agar from plates where bacteria were grown for five days); third set (**g**–**i**) antifungal activity by volatiles produced by SY1.

Three strains including SLB4, SLB6 and SY1 were found to be highly active in terms of plant growth promotion in rice and Bermuda grass seedlings. SY1 was found to possess the overall highest antifungal capacity based on our experiments. In antagonistic activity assays, SY1 strongly inhibited the tested fungal pathogens with 85.7% inhibition of *F. oxysporum*, 86.3% inhibition of *Curvularia* sp. and 82.1% inhibition of *Alternaria* sp. (Table 3, Figure 2). In agar diffusion assays, we found that diffused metabolite of SY1 in agar significantly inhibited several fungal pathogens (Figure 2, Table 3). In volatile antifungal compound assays, we found that SY1 produced volatile(s) that inhibited all tested fungi (Figure 2). Production of HCN was also indicated in cultures of SY1.

### 3.4. SY1 Exhibits Antifungal Activity in Grass Seedling Roots against Fusarium oxysporum

**Figure 3:**
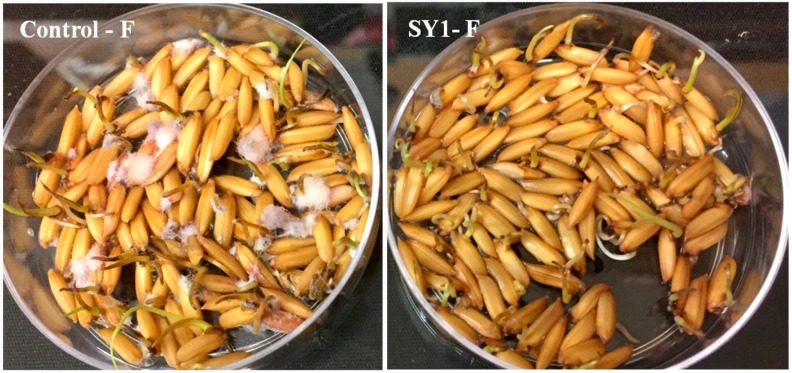

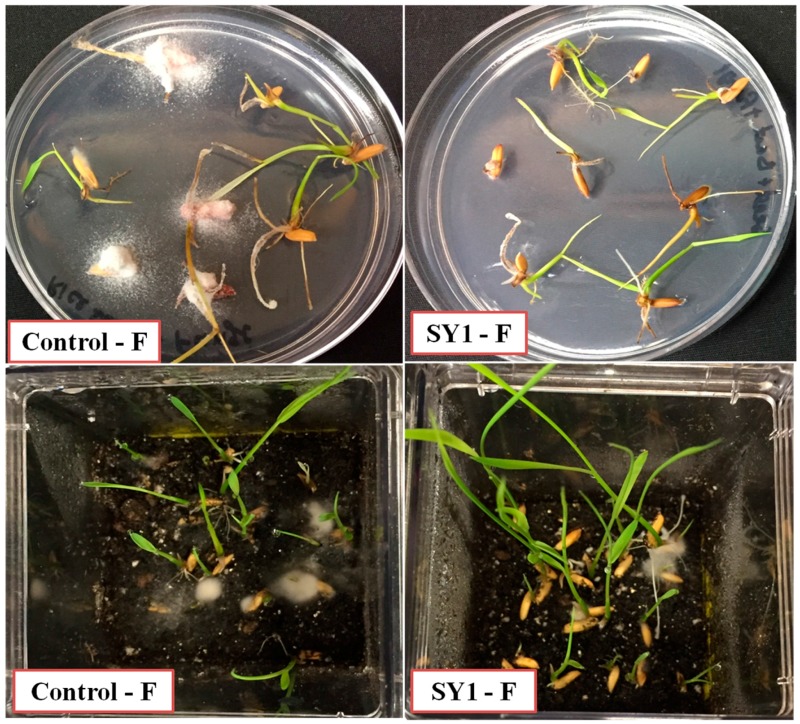
Inoculation of SY1 (*Pseudomonas* sp.) onto rice seeds, protecting rice seedlings from *Fusarium oxysporum* infection. First set is in Petri plates with water (three-days-old); second set in 0.7% agarose plates (eight-days-old) and third set in magenta boxes containing potting mix (15-days-old).

**Figure 4:**
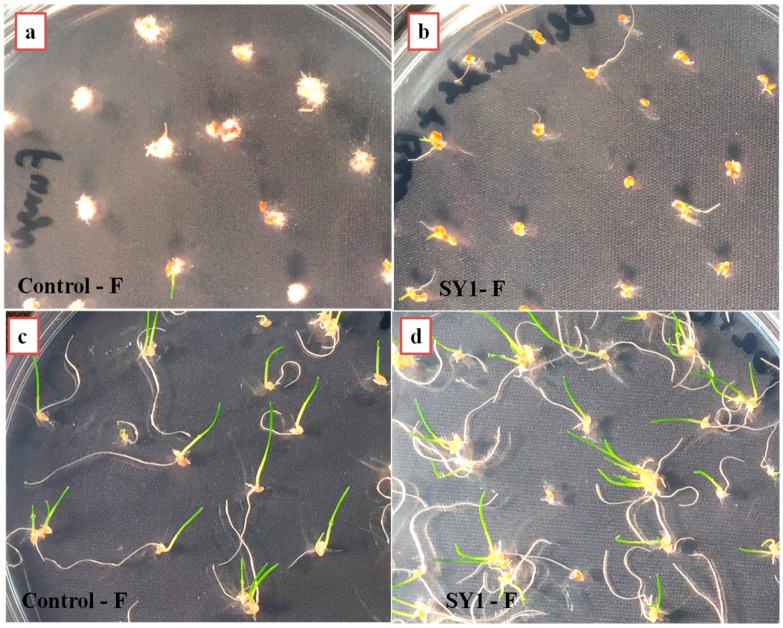
Protection of Bermuda grass (**a**,**b**) and annual bluegrass (**c**,**d**) seedlings on 0.7% agarose from *F. oxysporum* infection by SY1 isolate. More surviving seedlings are evident in (**b**,**d**) where bacterium SY1 and the fungus were present, than in (**a**,**c**) where only the fungus was present.

**Figure 5:**
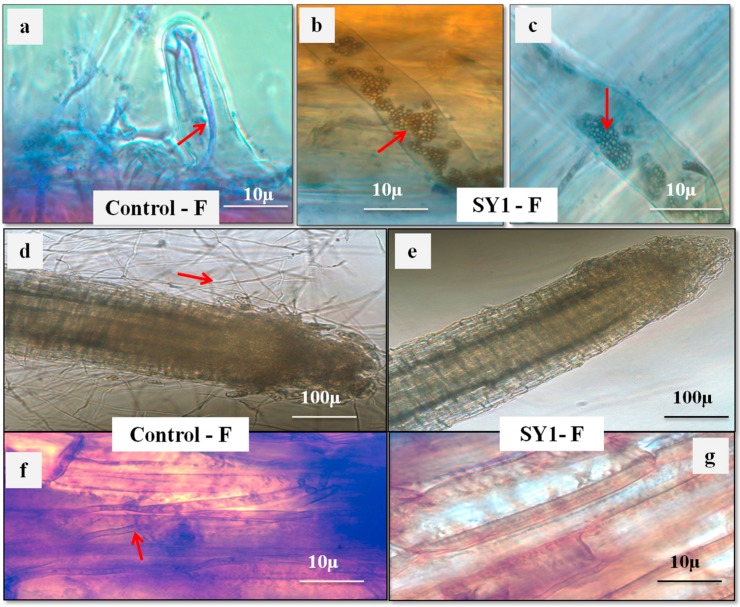
Microscopic view of roots (stained with diaminobenzidine tetrachloride (DAB)); In Bermuda grass (**a**–**c**): Where control (**a**) is infected with *F. oxysporum* (arrow indicates hypha within root hair); SY1-treated (**b**,**c**) found free of hyphae, but bacterial L-forms are visible inside root hairs (arrows). In annual bluegrass (**d**–**g**): (**d**,**f**) are control annual bluegrass root (**d**) and root parenchyma (**f**) colonized by fungus (arrows), and (**e**,**g**) are treated with SY1 and found free of infection by fungus *F. oxysporum*.

SY1-treated rice, Bermuda grass and annual bluegrass seeds were found to have reduced infection by *F. oxysporum.* As Table 4 and Figure 3 and Figure 4 show, SY1-treated seedlings were found to have reduced infection by *Fusarium* (18.75% for rice, 15% for Bermuda grass and 10% for annual bluegrass) compared to controls (51.50% for rice, 100% for Bermuda grass and 90% for annual bluegrass). When we observed the roots of annual bluegrass and Bermuda grass under a microscope after staining with DAB, we found that the SY1 treatment significantly checked the growth of fungal mycelium on the surfaces of and within tissues of roots (Figure 5). Intercellular and intracellular bacteria were also observed in treated seedling roots. 

**Figure 6:**
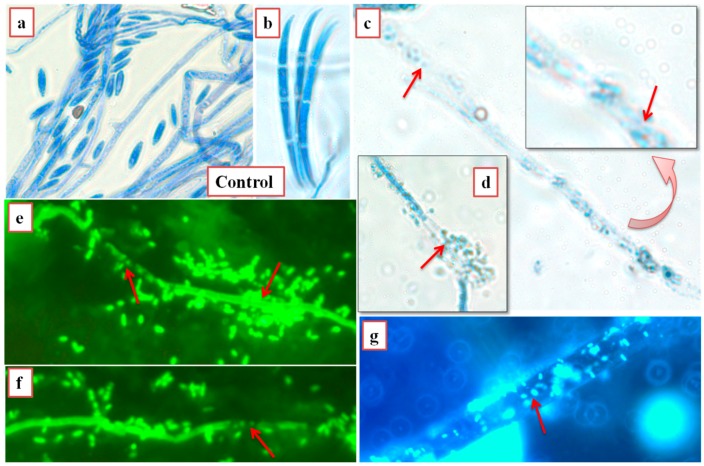
*F. oxysporum* from rice seedlings (magnification = 1000×). Where (**a**,**b**) are controls (without SY1 bacterial treatment) showing mycelium and conidia without bacteria, and (**c**–**g**) are treated with the bacterium SY1 showing degrading mycelium (arrows). ((**a**–**d**) are stained with cotton blue and (**e**–**g**) are stained with SYTO@13).

In interactions of fungi and bacteria on 0.7% agarose plates with seedlings, we observed that bacteria surrounded the hyphae and inhibited growth of mycelium (Figure 6). The cytoplasm of hyphae and spores was found to constrict and fragment in the presence of bacteria. Similar effects were observed in hyphae and spores of all three fungi tested.

### 3.5. Screening for Antibiotic Genes in SY1

**Figure 7:**
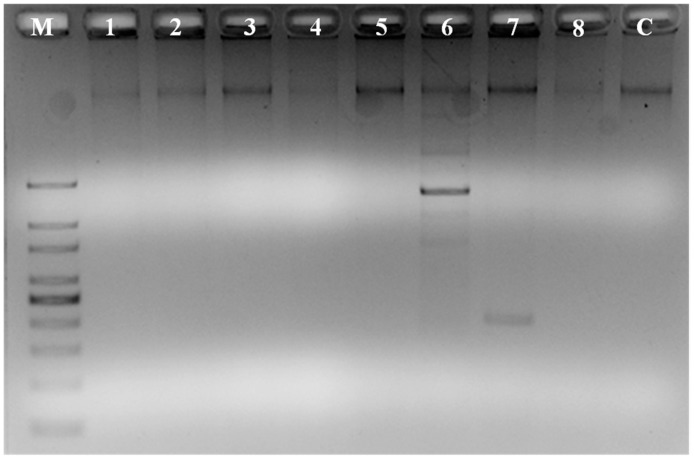
Polymerase chain reaction (PCR) products of different antibiotic genes amplified. Where M = marker, C = control, 1 = PHZ, 2 = phzCD, 3 = phze, 4 = phzF (1-4 related with phenezine synthesis), 5 = phlD-2,4-Diacetylphloroglucinol, 6 = prnD-pyrrolnitrin, 7 = hcnBC-hydrogen cyanide, 8 = PLTC-pyoleutirin. Lanes 6 and 7 suggest genes for pyrrolnitrin and HCN synthesis, respectively.

Out of the eight genes screened, two genes, including prnD (pyrrolnitrin) and hcnBC (hydrogen cyanide), were amplified successfully from the genome of SY1 (Figure 7). The amplified PCR product sizes correspond to sizes of respective genes. Gene prnD was found around ~800 bps, and hcnBC was around ~500 bps.

### 3.6. MALDI-TOF Analysis of SY1

MALTI-TOF analysis did not reveal conclusively the presence of lipopeptides in cultures of SY1. 

## 4. Discussion

Plants have evolved, in part, through continuous interaction with microbes, and it is becoming evident that some microbes play important roles in increasing the capacities of plants to survive and adapt [4,22,23]. Many plants carry microbes on or within their seeds, and these microbes may be critical for seedling survival. Plants may also recruit microbes from soils around roots [24,25]. The use of microbes that are symbiotic with plants to promote plant growth and control disease is potentially cost effective and may result in reductions in the use of synthetic fertilizers and pesticides to cultivate crop plants [5]. In the present study, we evaluated nine bacteria isolated from seeds of *Phragmites* [9], and of these, five were found to stimulate growth of rice and other grass seedlings (Table 1 and Table 2). Three bacteria (including, SLB4 (*P. fluorescens*), SLB6, (*Pseudomonas* sp.) and SY1 (*Pseudomonas* sp.)) were found to be especially potent (Figure 1). Seedling growth promotion including root hair formation and branching may be attributed to signaling through reactive oxygen species (ROS) and/or auxins [8,25,26] or could be induced by nutrient mobilization by bacteria during seedling development [27]. All tested bacteria were found to be positive for phosphate solubilization (Table 1). Plant roots also take up symbiotic bacteria into periplasmic spaces of root cells and degrade them oxidatively in the ‘rhizophagy symbiosis’ to supplement soil nutrient supplies [9,28]. We observed spherical wall-less bacterial ‘L-forms’ inside root hair cells and root parenchyma cells after reactive oxygen staining with DAB, as is common for intracellular bacteria in the rhizophagy symbiosis [9,25,28]. In terms of nutrient supply, root hairs and root parenchyma cells could be crucial regions for the internal localization of bacteria because this allows nutrients from the bacteria to be extracted, absorbed and translocated to other parts of the plant [13,29]; further, once internalized into the root tissues, the microbes themselves may move to other parts of the plant via the xylem [4].

*Pseudomonas* sp. (strain SY1) demonstrated strong antifungal activity in agar diffusion and in production of antifungal volatiles against *F. oxysporum*, *Curvularia* sp. and *Alternaria* sp. (Table 3, Figure 2). When we used strain SY1 as a biocontrol against *F. oxysporum* on rice, Bermuda grass and annual bluegrass during seed germination and early seedling development, we found that SY1 significantly reduced the infection of seedlings, effectively protecting them from disease (Table 4, Figure 3 and Figure 4). Similar results were observed in magenta boxes with potting mix (Figure 3). In microscopic examination, highly reduced or no infection by *Fusarium* was observed in roots of rice, Bermuda grass and annual bluegrass treated with SY1, in comparison to the non-treated controls (Figure 5). From microscopic observation of fungal mycelium with bacteria, we found that SY1 surrounded the hyphae and appeared to degrade it (Figure 6). In presence of SY1, fungal hyphal and conidial cytoplasm was found to constrict and break into pieces. Antifungal activity of SY1 (*Pseudomonas* sp.) may be in part due to production of antifungal compounds, including HCN or by other secondary metabolites. Various *Pseudomonas* species are known to inhibit fungal pathogens using HCN and other antimicrobial chemicals. Pyrrolnitirin is a chemical previously reported from *Pseudomonas* spp. with strong antifungal activity against *Fusarium* sp. [5,19,30,31]. In the present study, we found that the genes involved in pyrrolnitirin synthesis and HCN production may be present in the genome of SY1 based on amplification of genes of the appropriate size using specific primers. SY1 was confirmed to produce HCN on King’s B medium. Our MALTI-TOF analysis for lipopeptides was inconclusive for lipopeptide production. Lipopeptides are often found in Gram-positive bacteria and rarely reported from Gram-negative bacteria like *Pseudomonas* [32]. Whether SY1 produces lipopeptides will require further examination. Fernando et al. [33] reported antifungal volatiles from *Pseudomonas* spp. In our antifungal test (Figure 2g–i), we found that SY1 volatiles inhibited radial growth of *F. oxysporum*. The volatiles produced by SY1 were not identified. Hydrogen cyanide could account for the volatile inhibitory activity of SY1. 

Endophytyic bacteria have been reported to protect plants indirectly by inducing host defense genes. Gond et al. [11] found that an endophyte of corn, *Bacillus amyloliquefaciens*, induced expression of defense genes, including PR1 and PR4, which act against fungal pathogens. Endophytic bacteria also enhance the production of reactive oxygen species that could directly or indirectly defend plants from pathogen infection. Generally, roots with bacterial endophytes stained darker brown with DAB (by reactive oxygen staining) than plant roots without bacterial endophytes. Pathogens require specific signaling molecules (quorum sensing molecules) for infection of the host plant. Plant-associated endophytes may change or degrade these quorum sensing molecules and suppress pathogenicity [24]. Quorum-quenching lactonases from *P. fluorescens* have been found to largely protect potato tubers against *Pectobacterium carotovorum* soft rot [34].

Regardless of the exact mechanisms of plant growth promotion and biocontrol activity, here we report the successful application of seed-associated endophytic bacteria from invasive *Phragmites* to promote seedling growth and prevent disease incited by *F. oxysporum* in rice, Bermuda grass and annual bluegrass. Bacterial biocontrol agents may be applied to replace chemical fertilizers and pesticides to support sustainable agriculture. More focused work and field trials are needed to apply these microbes under agricultural conditions. 

## Figures and Tables

**Table 1:** Screening of bacteria on rice for effects on seedling development and phosphate solubilization activity ^t^.

Treatment	Geotropic Response (%)	Root Length (Mean ± SD cm)	Root Hairs and Branching *	Shoot Length (Mean ± SD cm)	Phosphate Solubilization
No bacteria	10	1.46 ± 0.53	+	1.45 ± 0.51	−
West9	20	2.28 ± 0.86	++	2.13 ± 0.75	+
SLB4	40	2.39 ± 0.91	+++	2.37 ± 0.90	+
Microbac	20	2.30 ± 0.84	++	2.18 ± 0.79	+
SY1	25	2.23 ± 083	+++	2.19 ± 0.76	+
SY5	10	1.47 ± 0.62	None	1.47 ± 0.65	+
SY4	0.0	1.37 ± 0.60	None	1.44 ± 0.63	+
SLB6	25	1.33 ± 0.56	++	1.25 ± 0.42	+
RiY3	0.0	2.50 ± 0.73	None	2.68 ± 0.74	+
ROLB13w	10	0.93 ± 0.58	None	1.03 ± 0.53	+

^t^ Seedlings (10 seedlings each treatment) were observed after seven days of incubation on agarose plates;.* +++ = very good development, ++ = good development, + = poor development.

**Table 2:** Screening of bacteria on Bermuda grass for effects on seedling development ^t^.

Treatment	Geotropic Response (%)	Root Length (Mean ± sd cm)	Root Hairs and Branching *	Shoot Length (Mean ± sd cm)
No bacteria	35	1.53 ± 0.35	+	1.58 ± 0.37
West9	35	1.87 ± 0.58	+++	1.88 ± 0.59
SLB4	50	2.23 ± 0.58	+++	2.43 ± 0.92
Microbac	40	1.65 ± 0.83	+	1.36 ± 0.67
SY1	40	2.45 ± 0.55	+++	2.43 ± 0.70
SLB6	60	1.99 ± 0.59	+++	2.24 ± 0.47

^t^ Seedlings (10 seedlings each treatment) were observed after seven days of incubation on 0.7% agarose plates; * +++ = very good development, ++ = good development, + = poor development.

**Table 3:** Percent inhibition of fungal pathogens *Fusarium oxysporum, Curvularia* sp. and *Alternaria* sp. by SY1 in antagonism and agar diffusion assays.

Treatments	% Inhibition		
	*F. oxysporum*	*Curvularia* sp.	*Alternaria* sp.
Antagonism	85.71	86.33	82.14
Agar diffusion	52.38	63.63	55.55

**Table 4:** Percent of seedlings of rice, Bermuda grass and annual bluegrass showing infection by *F. oxysporum* after five days of treatment with SY1 and untreated controls.

Treatments	% Infection of Seedlings		
	Rice	Bermuda Grass	Annual Bluegrass
Control (Fungi)	51.50	100.00	90.00
Treatment (SY1 + Fungi)	18.75	15.00	10.00
